# SmartSurf^ACE^ transepithelial photorefractive keratectomy with mitomycin C enhancement after small incision lenticule extraction

**DOI:** 10.1186/s40662-021-00254-2

**Published:** 2021-09-01

**Authors:** Amr A. Gab-Alla

**Affiliations:** Faculty of Medicine, Ophthalmology Department, Suez Canal University, Ring Road, Ismailia, Egypt

**Keywords:** Transepithelial PRK, Mitomycin C, Refractive enhancement, Small incision lenticule extraction, Smart pulse technology, SmartSurface procedure

## Abstract

**Background:**

To evaluate predictability, stability, efficacy, and safety of transepithelial photorefractive keratectomy (TPRK) using smart pulse technology (SPT) (SmartSurface procedure) of Schwind Amaris with mitomycin C for correction of post small incision lenticule extraction (SMILE) myopic residual refractive errors.

**Method:**

This study is a prospective, non-comparative case series conducted at a private eye centre in Ismailia, Egypt, on eyes with post-SMILE myopic residual refractive errors because of undercorrection or suction loss (suction loss occurred after the posterior lenticular cut and the creation of side-cuts; redocking was attempted, and the treatment was completed in the same session with the same parameters) with myopia or myopic astigmatism. The patients were followed up post-SMILE for six months before the SmartSurface procedure, and then they were followed up for one year after that. TPRK were performed using Amaris excimer laser at 500 kHz. The main outcomes included refractive predictability, stability, efficacy, safety and any reported complications.

**Results:**

This study included 68 eyes of 40 patients out of 1920 total eyes (3.5%) with post-SMILE technique myopic residual refractive errors. The average duration between the SMILE surgery and TPRK was 6.7 ± 0.4 months (range 6 to 8 months). The mean refractive spherical equivalent (SE) was within ± 0.50 D of plano correction in 100% of the eyes at 12 months post-TPRK. Astigmatism of < 0.50 D was achieved in 100% of the eyes. The mean of the residual SE error showed statistically significant improvement from preoperative − 1.42 ± 0.52 D to 0.23 ± 0.10 D (*P* < 0.0001). Uncorrected distance visual acuity (UDVA) (measured by Snellen's chart and averaged in logMAR units) was improved significantly to 0.1 ± 0.07 (*P* < 0.0001). UDVA was 0.2 logMAR or better in 100% of the eyes, 0.1 logMAR or better in 91.2% of the eyes, and 0.0 logMAR in 20.6% of the eyes. Corrected distance visual acuity (CDVA) remained unchanged in 79.4% of eyes. 14.7% of eyes gained one line of CDVA (Snellen). 5.9% of eyes gained two lines of CDVA (Snellen).

**Conclusion:**

Transepithelial photorefractive keratectomy using smart pulse technology with mitomycin C enhancement after SMILE is a safe, predictable, stable, and effective technique.

## Introduction

Small incision lenticule extraction (SMILE) technique was introduced to preserve biomechanical properties of the cornea, reduce injury to corneal nerves, which results in earlier recovery of postoperative corneal sensitivity, and thus lower incidence of dry eye when compared with laser-assisted in situ keratomileusis (LASIK) [1]. In several studies, the SMILE technique was shown to be predictable, safe, and effective in treating myopia and myopic astigmatism [2].

The most common complication of any refractive procedure is residual refraction. Overcorrection, undercorrection, induced or residual astigmatism are all included; however, refractive regression is characterised as the gradual, partial, or complete loss of initial correction, limiting the predictability, efficiency, and long-term stability of this refractive procedure [3, 4].

Retreatment rates after SMILE have not been extensively reported [1]; Reinstein et al. [5] reported a 4% enhancement rate after low myopic treatment. Hjortdal et al. [6] reported that 20% of the patients have ≥ 0.5 D and 6% have ≥ 1.0 D of myopia at the 3rd month post-SMILE in eyes with preoperative mean refractive spherical equivalent (SE) − 7.19 ± 1.30 D. It cannot be totally ruled out that some SMILE patients may have a residual refractive error from initial undercorrection, regression, or induced astigmatism [7]. If the residual refractive error is significant enough, these patients may require further refractive correction or enhancement. Though flap re-lifting and refractive enhancement can be easily done post-LASIK, the absence of a flap in SMILE is a unique challenge when refractive enhancement is indicated.

There are many alternatives to choose to correct post-SMILE residual refractive errors or post-SMILE myopic regression [8]. Photorefractive keratectomy (PRK) benefits from not making a corneal flap and preserving corneal stromal tissue, and thus avoids the potential risk of postoperative keratectasia [9]. PRK techniques have improved in recent years [10]. A new technique TPRK, has been introduced as an alternative to conventional PRK. This avoided the need for alcohol epithelial debridement or mechanical removal of the epithelium during PRK. In fact, TPRK requires only a one-step removal of the epithelium. The use of TPRK results in reduced operating time, no instrument contact with the cornea, decreased postoperative discomfort, faster healing and visual recovery when compared with traditional PRK [10, 11]. SmartSurface treatment (SCHWIND eye-tech-solutions GmbH, Kleinostheim, Germany) is a combination of TPRK using the smart pulse technology (SPT) [12]. With the SPT profile, the volume is based on a curved corneal surface by a fullerene structure, meaning that every ablation point is equidistant [12]. However, there is a deficiency of data on the safety, efficacy, and refractive outcomes of SmartSurface to enhance post-SMILE residual errors. Therefore, this study aimed to evaluate the predictability, stability, efficacy, and safety of SmartSurface with mitomycin C (MMC) to correct post-SMILE myopic residual refractive errors.

## Methods

This study is a prospective, non-comparative case series of 68 eyes in 40 patients conducted at a private eye centre in Ismailia, Egypt. All patients provided written informed consent for the procedure and inclusion in this study. This study adheres to the tenets of the Declaration of Helsinki. It was reviewed and approved by the Faculty of Medicine, Suez Canal University Research Ethics Committee. The patients were operated on, examined, and followed up by one surgeon (AAG).

Patients underwent SmartSurface treatment, which was a combination of TPRK using the SPT. Inclusion criteria were patients with post-SMILE myopic residual refractive errors because of undercorrection or suction loss (suction loss occurred after the posterior lenticular cut and the creation of side-cuts; redocking was attempted, and the treatment was completed in the same session with the same parameters) including residual and/or consecutive astigmatism ≥ 0.5 D. The patients were followed up post-SMILE for six months before TPRK. Other inclusion criteria were patients with intraocular pressure (IOP) < 21 mmHg, sufficient corneal thickness to leave > 60% of the original total corneal thickness, and regular corneal topography pattern (Sirius, CSO, Florence, Italy). Patients were excluded if they had consecutive hyperopia with or without residual or consecutive astigmatism, diabetes mellitus, autoimmune diseases, corneal scars, or ectasia. Patients with insufficient follow-up were excluded from the study.

### SMILE technique

Preoperatively, patients experienced standard eye examinations including slit-lamp examination, indirect fundoscopy, and refraction (cycloplegic and manifest), IOP, and biomechanical properties of the cornea (corneal hysteresis (CH) and corneal resistance factor (CRF)) by ocular response analyser II (ORA, Reichert, Depew, New York). Uncorrected distance visual acuity (UDVA) and corrected distance visual acuity (CDVA) measured by Snellen's chart (logMAR units) was recorded. SMILE technique was performed using refractive lenticule extraction (ReLEx®) on the VisuMax 500 kHz femtosecond laser system (Carl Zeiss Meditec, Jena, Germany). Attempted cap thickness was set at 110 ± 10 μm (to ensure that the residual stromal bed was maintained at > 60% of the corneal thickness) and exceeded the lenticule diameter by 1.0 to 2.0 mm. The size of the lenticule ranged from 6.0 to 7.0 mm, with no transition zone for spherical errors and a 0.1 mm transition zone for astigmatism. It was adjusted according to the mesopic pupil diameter of the patients. The entering incision varied from 3.0 to 4.0 mm. Postoperative follow-up visits were on the 1st day, 1st week, 1st month, 3rd month, and 6th month. Follow-up visits included full ophthalmic examinations, cycloplegic and manifest refraction, corneal topography, UDVA and CDVA, biomechanical properties of the cornea by ocular response analyser II, and slit-lamp examination.

### SmartSurface TPRK with mitomycin C

All the treatments were done using Amaris excimer laser at 500 kHz with plano target, aspheric, and non-wavefront-guided profiles. TPRK were performed only when the estimated residual stromal thickness was greater than 300 μm. After povidone-iodine scrub of the eyelids and application of skin topical anaesthesia with 0.4% benoxinate hydrochloride drops at 5-min intervals, a wet sponge by balanced salt solution (BSS) was used before laser ablation to uniformly wipe the corneal surface, preventing uneven wetting and, accordingly, uneven ablation. The planning software calculated the size of the optimal transition zone for each treatment, depending on the preoperative refraction and optical treatment zone. The enhancement was ablated by TPRK mode using smart SPT (SmartSurface TPRK). The epithelial ablation profile software uses a central epithelium ablation of 55 µm at the centre and 65 µm at 4 mm. MMC (0.02%) (Kyowa-hakko Co. Ltd., Tokyo, Japan) was applied to the stromal bed for 30 s by a wet sponge. The surface was irrigated with BSS and dried. Then, a soft contact lens (Pure Vision, Bausch & Lomb) was added, and one drop of 0.3% Tobramycin and 0.1% Dexamethasone was instilled (Tobradex, Alcon laboratories). The patients were advised to use 0.5% moxifloxacin hydrochloride (Vigamox, Alcon laboratories) qid till re-epithelization, remove contact lens, and apply a combination of 0.3% topical Tobramycin and 0.1% Dexamethasone qid for the first week, then decrease to once a week, and 0.3% sodium hyaluronate drops (Systane ultra, Alcon laboratories) qid for 4 months.

Postoperatively, the patients were examined on the 1st day, 1st week, 1st, 3rd, 6th, and 12th month. Patients’ data were reported at 1, 3, 6, and 12 months postoperatively. Patients were assessed by complete ocular examinations, IOP, CH, CRF by ocular response analyser II, corneal topography, refraction (cycloplegic and manifest), CDVA and UDVA. Complications were recorded. According to Fante's classification [11], the corneal haze was graded on a scale from 0 to 4.

The main outcomes included refractive predictability, which was assessed by the percentage of eyes < 0.5 D of the target correction using the SE. Stability was assessed by comparing postoperative cycloplegic refractions SE at follow-up times. Efficacy was assessed by the percentage of postoperative UDVA to the preoperative CDVA and the efficacy index was the ratio of the mean postoperative UDVA to the mean preoperative CDVA. Postoperative safety was assessed by the percentage of the eyes that gained/lost lines compared with preoperative CDVA; the safety index was the ratio of the mean postoperative CDVA to the mean preoperative CDVA and reported complications.

### Statistical analysis

Data were coded, entered, and analysed using SPSS (version 25.0, IBM, Armonk, NY, USA). Baseline data of the study population were presented as mean values, standard deviations, and range. Predicted (attempted) postoperative SE refraction was calculated using simple regression analysis, while the mean error in the treatment was calculated as the difference between the attempted and achieved postoperative SE refraction up to the 12th month after surgery. Analysis of visual acuity results was performed by calculating the geometric mean with a standard deviation into logMAR format from Snellen examination results*. P*-value (using the Mann–Whitney U test, Kruskal–Wallis H, repeated measures ANOVA tests for statistical significance) of less than 0.05 was considered statistically significant. Graphs were created using either SPSS or Microsoft Excel 2016.

## Results

This study included 68 eyes of 40 patients out of 1920 total eyes (3.5%) with post-SMILE myopic residual refractive errors for refractive enhancement. TPRK treatment was bilateral in 28 (70%) patients and unilateral in 12 (30%) patients (8 patients in the right eye and 4 patients in the left eye). Twenty-eight eyes were of male patients and 40 eyes of female patients. The average duration between SMILE surgery and TPRK was 6.7 ± 0.4 months (range 6 to 8 months). The demographic data of the SMILE and TPRK groups are presented in Table 1. The mean optical zone size of SMILE treatment was 6.5 ± 0.1 mm (range 6.1 to 7.0 mm). 100% of eyes had a CDVA 0.2 logMAR or better, 88.2% of eyes had a CDVA of 0.1 logMAR or better, and 17.6% of eyes achieved 0.0 logMAR or better. Mean ablation depth was 28.13 ± 6.47 μm (range 15 to 38 μm), and the mean central corneal thickness 12 months after TPRK was 472.53 ± 15.62 μm (range 455 to 483 μm). The demographic data are presented in Table 1.

**Table 1:** Baseline characteristics of the study population

Parameter	SMILE group	TPRK group
Eyes		
Number	1920	68
Patients	960	40
Laterality (n, %)		
Right	960 (50%)	36 (53%)
Left	960 (50%)	32 (47%)
Age (years)		
Mean ± SD	25.0 ± 2.8	26.0 ± 4.2
Range	(21 to 37)	(21 to 35)
Sex (n, %)		
Male	326 (34%)	16 (40%)
Female	634 (66%)	24 (60%)
Refraction SE (D)		
Mean ± SD	− 5.50 ± 1.20	− 1.42 ± 0.52
Range	(− 2.50 to − 8.50)	(− 0.75 to − 2.50)
Sphere (D)		
Mean ± SD	− 4.30 ± 1.32	− 1.21 ± 1.10
Range	− 2.25 to − 7.75	− 0.50 to − 2.10
Cylinder (D)		
Mean ± SD	− 1.63 ± 0.82	− 0.57 ± 0.50
Range	− 0.75 to − 2.75	0.00 to − 1.50
CCT (μm)		
Mean ± SD	530.0 ± 10.2	491.0 ± 13.2
Range	(508 to 560)	(470 to 511)
K readings (D)		
Mean ± SD	43.0 ± 1.6	40.0 ± 1.3
Range	(42 to 46)	(38 to 41)
CDVA (logMAR)		
Mean ± SD	0.26 ± 0.08	0.14 ± 0.06
Range	(0.00 to 0.6)	(− 0.1 to 0.2)
CH		
Mean ± SD	10.40 ± 0.92	8.60 ± 1.56
Range	(9.7 to 13.1)	(7.8 to 11.2)
CRF		
Mean ± SD	10.20 ± 1.24	9.20 ± 1.66
Range	(9.6 to 13.3)	(8.3 to 11.5)

*SMILE* small incision lenticule extraction; *TPRK* transepithelial photorefractive keratectomy; *SD* standard deviation; *SE* spherical equivalent; *CCT* central corneal thickness; *K* keratometry; *CDVA* corrected distance visual acuity; *CH* corneal hysteresis; *CRF* corneal resistant factor; *D* diopter

### Refractive predictability

**Fig. 1:**
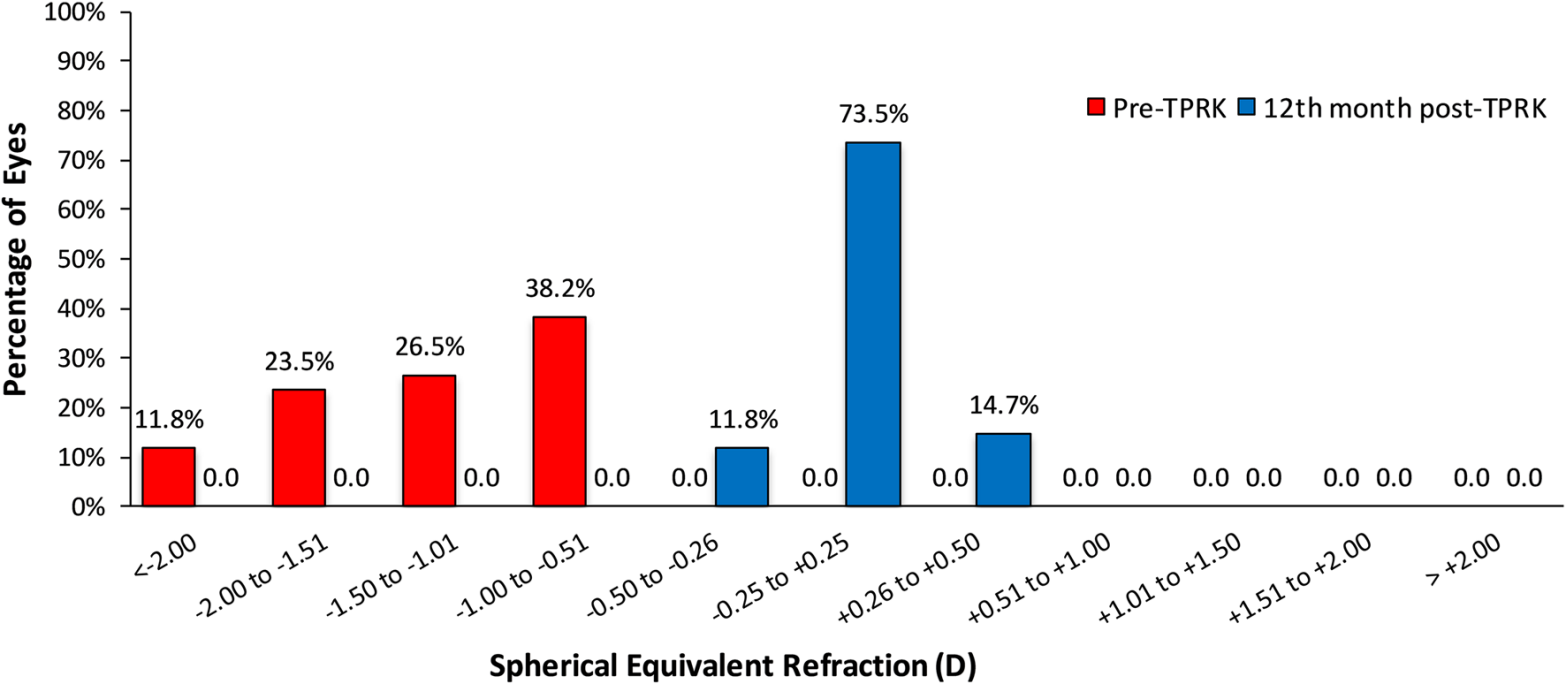
Distribution of post-TPRK mean refractive spherical equivalent (MRSE) at the 12th month postoperative (predictability)

At the 12th month post-TPRK, the mean refractive spherical equivalent (MRSE) was within ± 0.50 D of plano correction in 100% of eyes (68 eyes). The distribution of MRSE before and after TPRK can be found in Fig. 1. Pre-TPRK, 11.8% of eyes were within less than − 2.00 D, 23.5% of eyes were within − 2.00 to − 1.50 D, 26.5% of eyes were within − 1.50 to − 1.00 D and 38.2% of eyes were within − 1.00 to − 0.50 D of target refraction. After retreatment, 11.8% of eyes were within − 0.50 to + 0.25 D, 73.5% of eyes were within − 0.25 to + 0.25 D and 14.7% of eyes were within + 0.25 to + 0.50 D of target refraction.

**Fig. 2:**
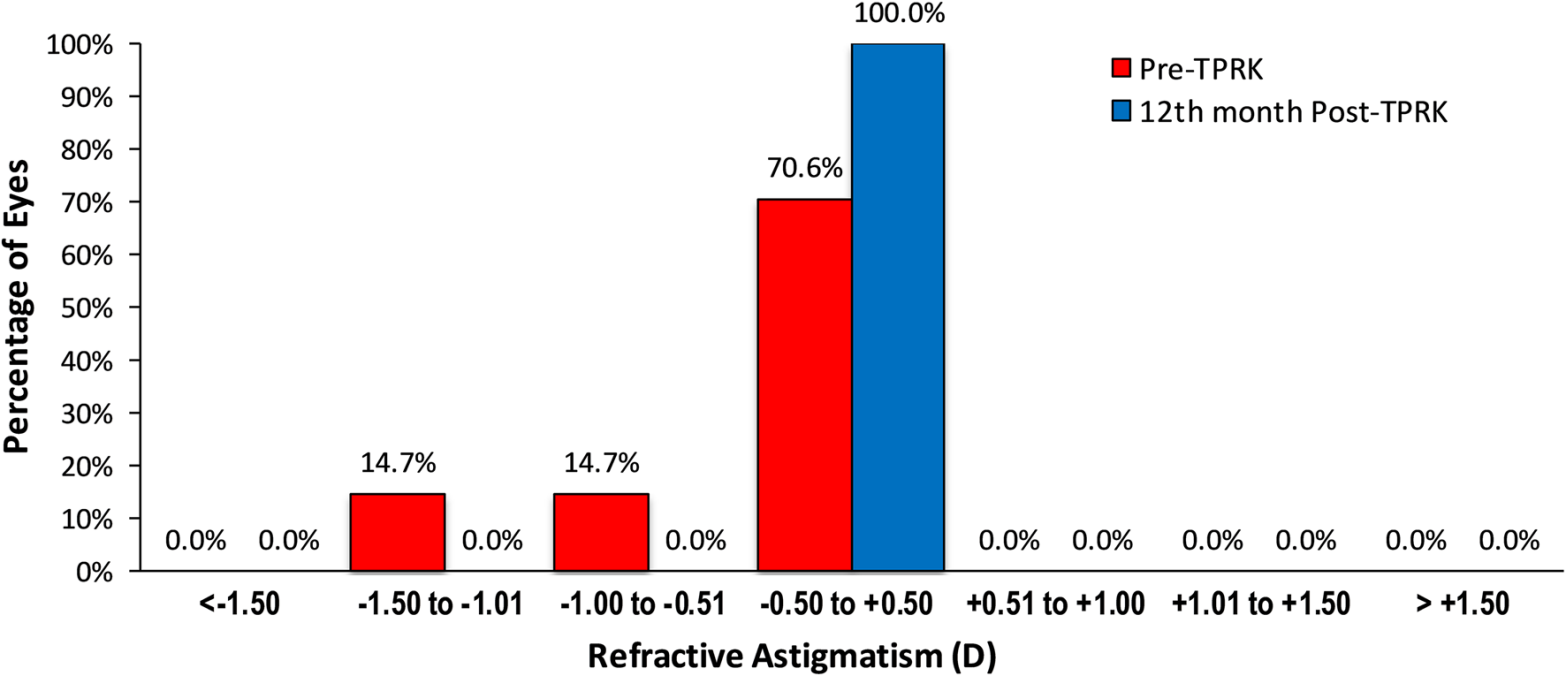
Distribution of post-TPRK refractive astigmatism at the 12th month postoperative. The number of patients within ± 0.50 D of astigmatism increased from 70.6 to 100%

**Fig. 3:**
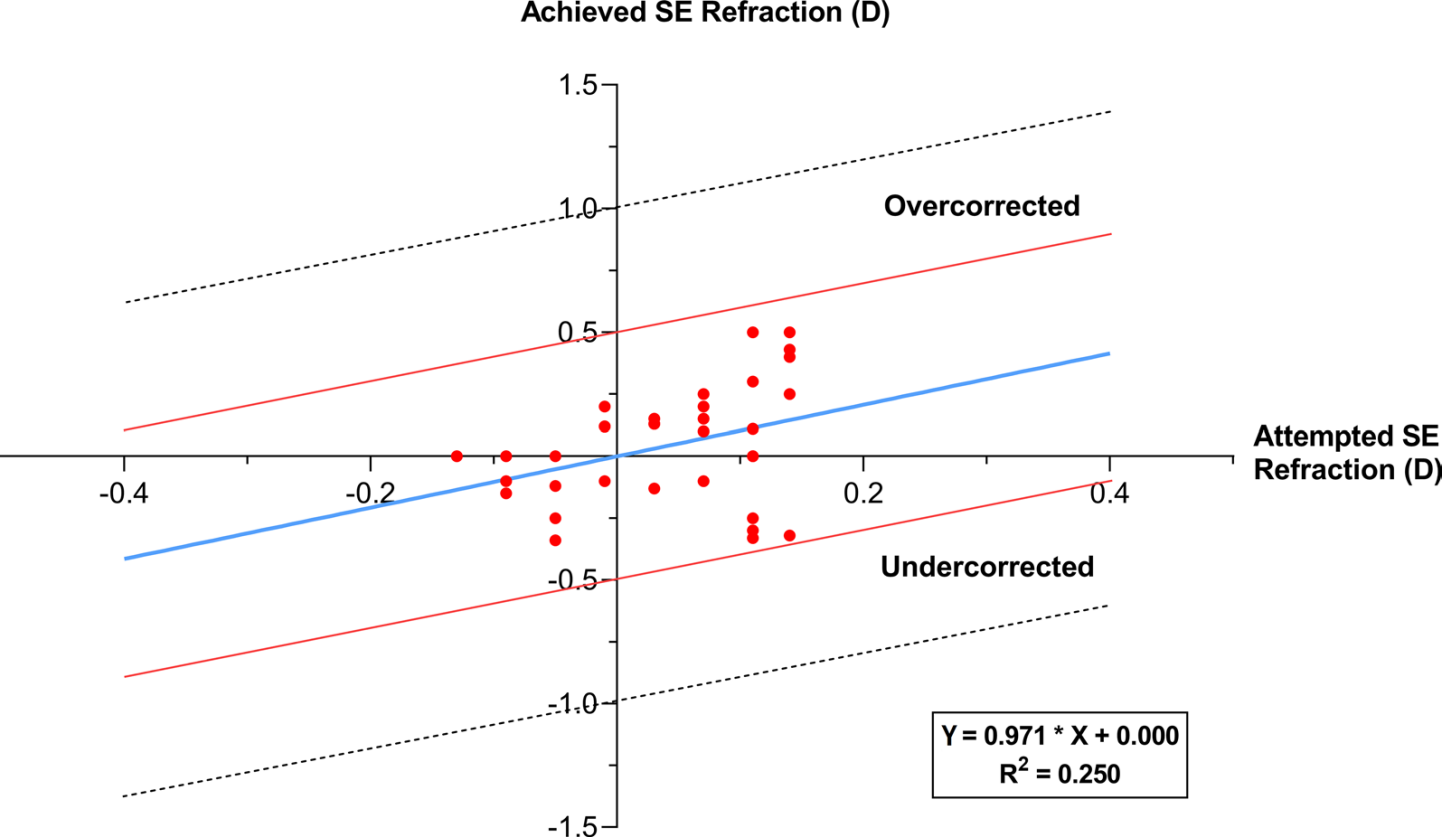
Scatterplot of the attempted spherical equivalent (SE) correction versus the achieved SE correction at 12th month post-TPRK

The mean refractive astigmatism was within 0.50 D and this was achieved in 100% of eyes (Fig. 2). Pre-TPRK, 14.7% of eyes were within − 1.50 to − 1.00 D, 14.7% of eyes were within − 1.00 to − 0.50 D, and 70.6% of eyes were within − 0.50 to + 0.50 D. Figure 2 shows the distribution of astigmatism before and after TPRK. Figure 3 shows the scatterplot of the attempted SE correction versus the achieved SE correction 12 months post-TPRK; it shows that MRSE was within ± 0.50 D of plano correction in 100% of eyes.

### Stability

**Fig. 4:**
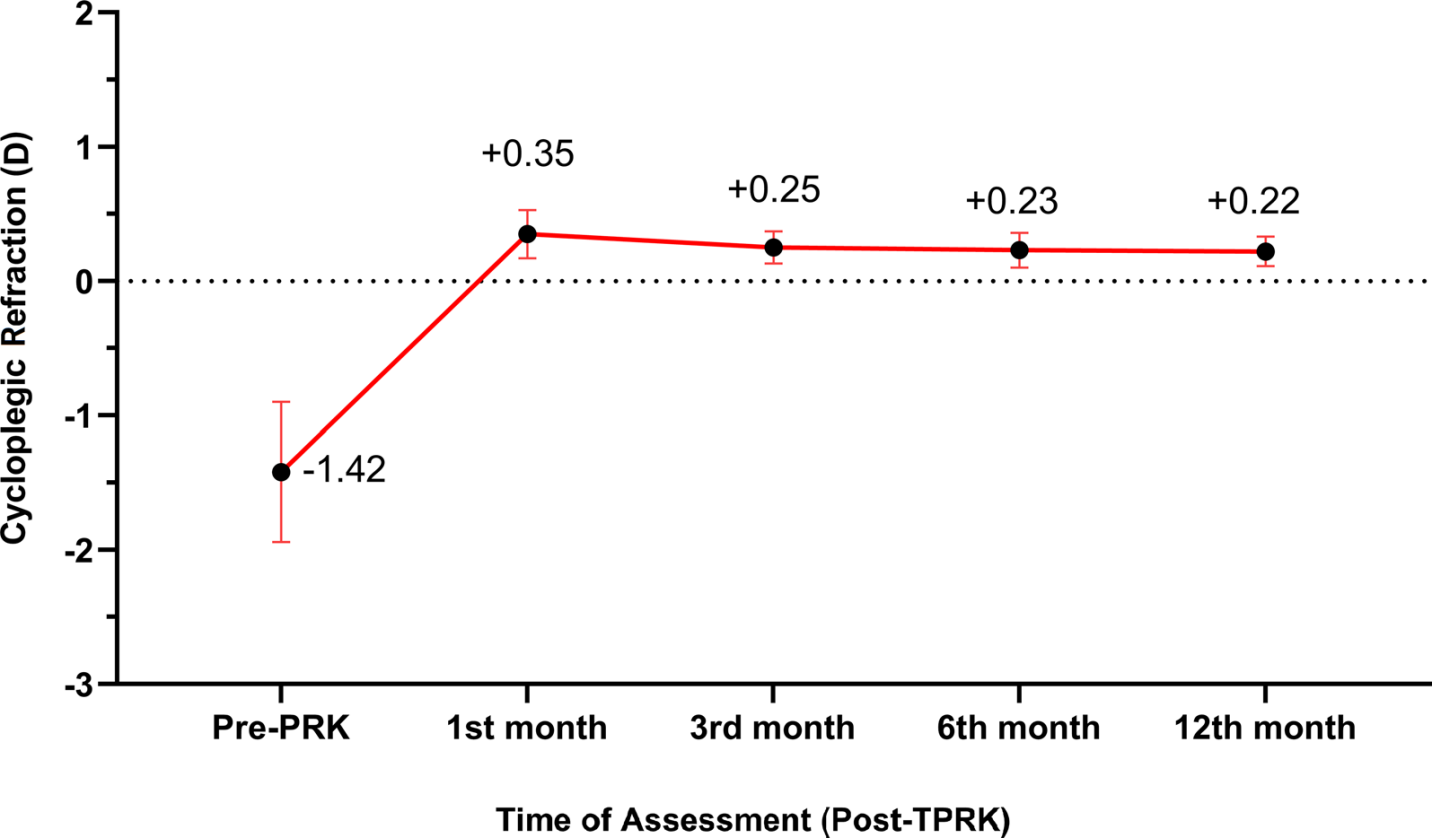
Post-TPRK changes in refractive stability

Post-TPRK data were reported at the 1st, 3rd, 6th, and 12th month. The mean of the residual MRSE showed statistically significant improvement (*P* < 0.0001) from pre-TPRK − 1.42 ± 0.52 D (range − 0.75 to − 2.50 D) to + 0.35 ± 0.18 D (range + 0.50 to − 0.50 D) at the 1st month, + 0.25 ± 0.27 D (range + 0.50 to − 0.25 D) at the 3rd month, + 0.23 ± 0.12 D (range + 0.25 to − 0.25 D) at the 6th month, and + 0.22 ± 0.11 D (range + 0.25 to 0.00 D) at the 12th month. There was a statistically significant improvement of the refraction up to the third post-TPRK month, then the stability of the refractions up to the 12th month (Table 2, Fig. 4).

**Table 2:** Post-TPRK cycloplegic refraction outcomes SE in diopters

Time of follow-up	Cycloplegic refraction (SE) Mean ± SD (Range)
Pre-TPRK	− 1.42 ± 0.52 (− 0.75 to − 2.50)
1st month post-TPRK	0.35 ± 0.18 (0.50 to − 0.50)
3rd month post-TPRK	0.25 ± 0.27 (0.50 to − 0.25)
6th month post-TPRK	0.23 ± 0.12 (0.25 to − 0.25)
12th month post-TPRK	0.22 ± 0.11 (0.25 to 0.00)
*P-*value	< 0.0001*

*TPRK* transepithelial photorefractive keratectomy; *SD* standard deviation; *SE* spherical equivalent

*Statistically significant

### Visual acuity and efficacy

Post-TPRK, mean UDVA (logMAR) significantly improved at the 1st, 3rd, 6th, and the 12th month to 0.15 ± 0.08 (range 0.25 to − 0.10), 0.10 ± 0.10 (range 0.2 to − 0.1), 0.10 ± 0.07 (range 0.18 to − 0.10), and 0.10 ± 0.06 (range 0.17 to − 0.10), respectively (Table 3). The efficacy index was 1.01 ± 0.10, 1.03 ± 0.13, 1.04 ± 0.15, and 1.04 ± 0.17, respectively.

**Table 3:** Post-TPRK logMAR of uncorrected distance visual acuity (UDVA)

Time of follow-up	UDVA (logMAR) Mean ± SD (Range)
Pre-TPRK	0.62 ± 0.16 (0.3 to 1.0)
1st month post-TPRK	0.15 ± 0.08 (0.25 to − 0.10)
3rd month post TPRK	0.10 ± 0.10 (0.2 to − 0.1)
6th month post TPRK	0.10 ± 0.07 (0.18 to − 0.10)
12th month post TPRK	0.10 ± 0.06 (0.17 to − 0.10)
*P-*value	< 0.0001*

*TPRK* transepithelial photorefractive keratectomy; *SD* standard deviation; *UDVA* uncorrected distance visual acuity

*Statistically significant

**Fig. 5:**
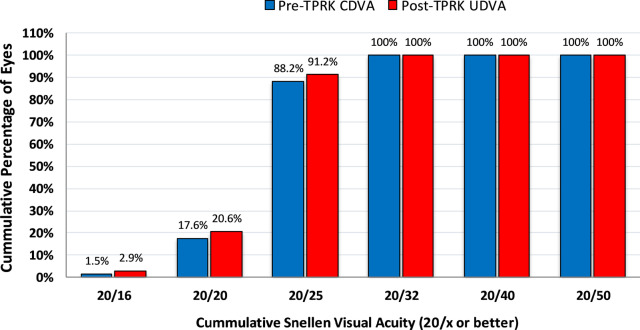
Comparison of pre-TPRK CDVA to post-TPRK UDVA at the 12th month postoperative (efficacy)

All patients had a significant improvement in UDVA after the TPRK at each point of follow up (*P* < 0.0001, Fig. 5). At the 12th month post-TPRK, UDVA was 0.2 logMAR or better in 100% of the eyes, 0.1 logMAR or better in 91.2% of the eyes, and 0.0 logMAR in 20.6% of the eyes.

### Safety

**Fig. 6:**
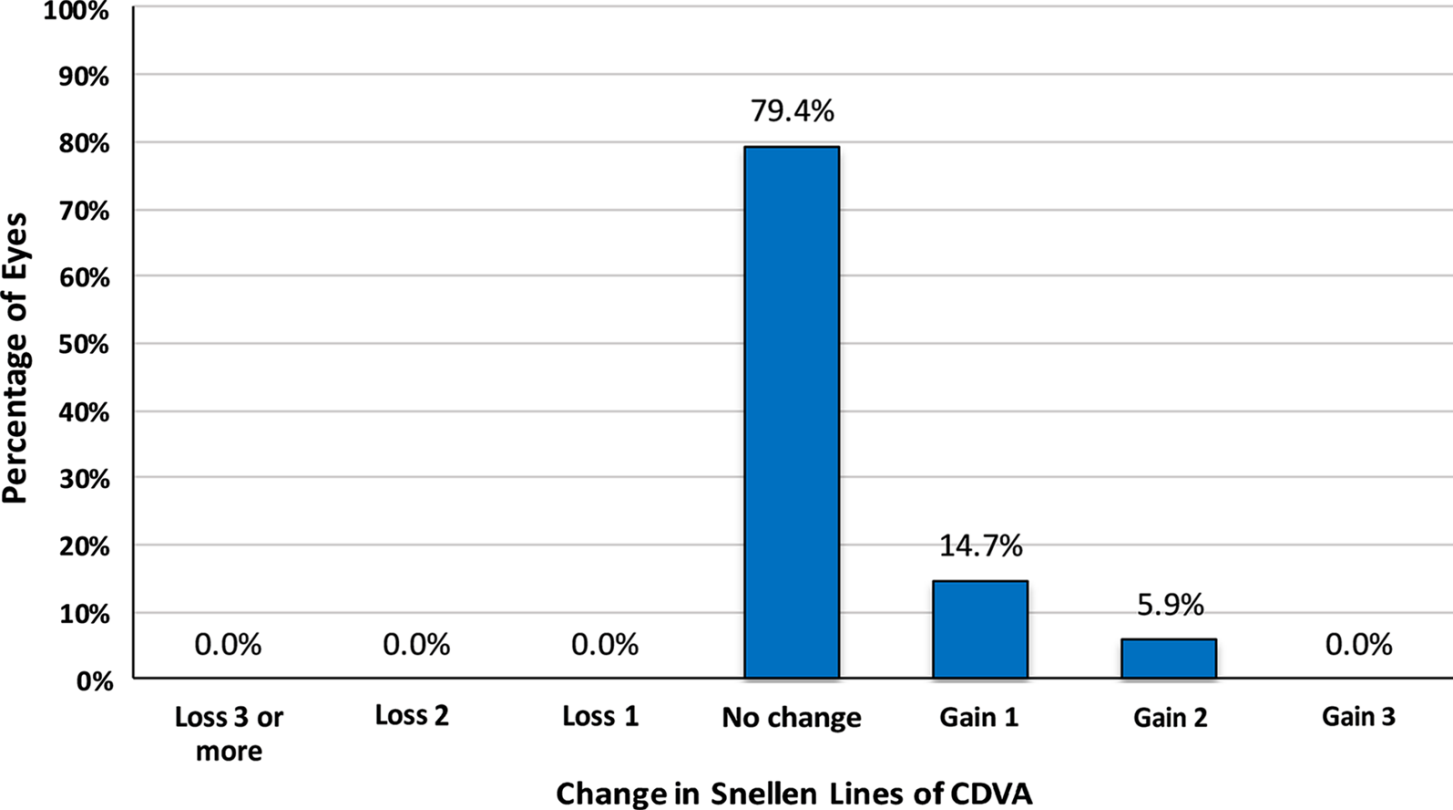
Changes in lines of CDVA at the 12th month post-TPRK (safety)

The safety index was 1.00 ± 0.09, 1.01 ± 0.04, 1.02 ± 0.02, and 1.02 ± 0.03 at the 1st, 3rd, 6th, and 12th month, respectively. The safety of the treatment assessed at the 12th month of follow-up, found that CDVA remained unchanged in 79.4% of eyes (n = 54). Ten eyes (14.7%) gained one line of CDVA (Snellen). Four eyes (5.9%) gained two lines of CDVA (Snellen). No eye had lost line of CDVA (Fig. 6).

### Corneal hysteresis and corneal resistance factor

The pre-TPRK mean CH was 8.60 ± 1.56 (range 7.80 to 11.20). Post-TPRK mean CH at the 1st, 3rd, 6th, and 12th month was 8.49 ± 1.50 (range 7.36 to 10.84), 8.48 ± 1.70 (range 7.36 to 10.84), 8.47 ± 1.60 (range 7.30 to 10.70), and 8.46 ± 1.57 (range 7.29 to 10.60), respectively. There was no significant change in CH during the follow-up period (*P* = 0.972, Table 4).

**Table 4:** Post-TPRK changes in corneal biomechanical properties (CH and CRF)

Time of follow-up	CH Mean ± SD (range)	CRF Mean ± SD (range)
Pre-PRK	8.60 ± 1.56 (7.80 to 11.20)	9.20 ± 1.66 (8.30 to 11.50)
1st month post-TPRK	8.49 ± 1.50 (7.36 to 10.84)	9.05 ± 1.80 (7.38 to 10.90)
3rd month post-TPRK	8.48 ± 1.70 (7.36 to 10.84)	9.01 ± 1.40 (7.38 to 10.90)
6th month post-TPRK	8.47 ± 1.60 (7.30 to 10.70)	8.97 ± 1.50 (7.28 to 10.90)
12th month post-TPRK	8.46 ± 1.57 (7.29 to 10.60)	8.96 ± 1.50 (7.29 to 10.87)
*P-*value	0.972	0.823

*TPRK* transepithelial photorefractive keratectomy; *SD* standard deviation; *CH* corneal hysteresis; *CRF* corneal resistance factor

**Fig. 7:**
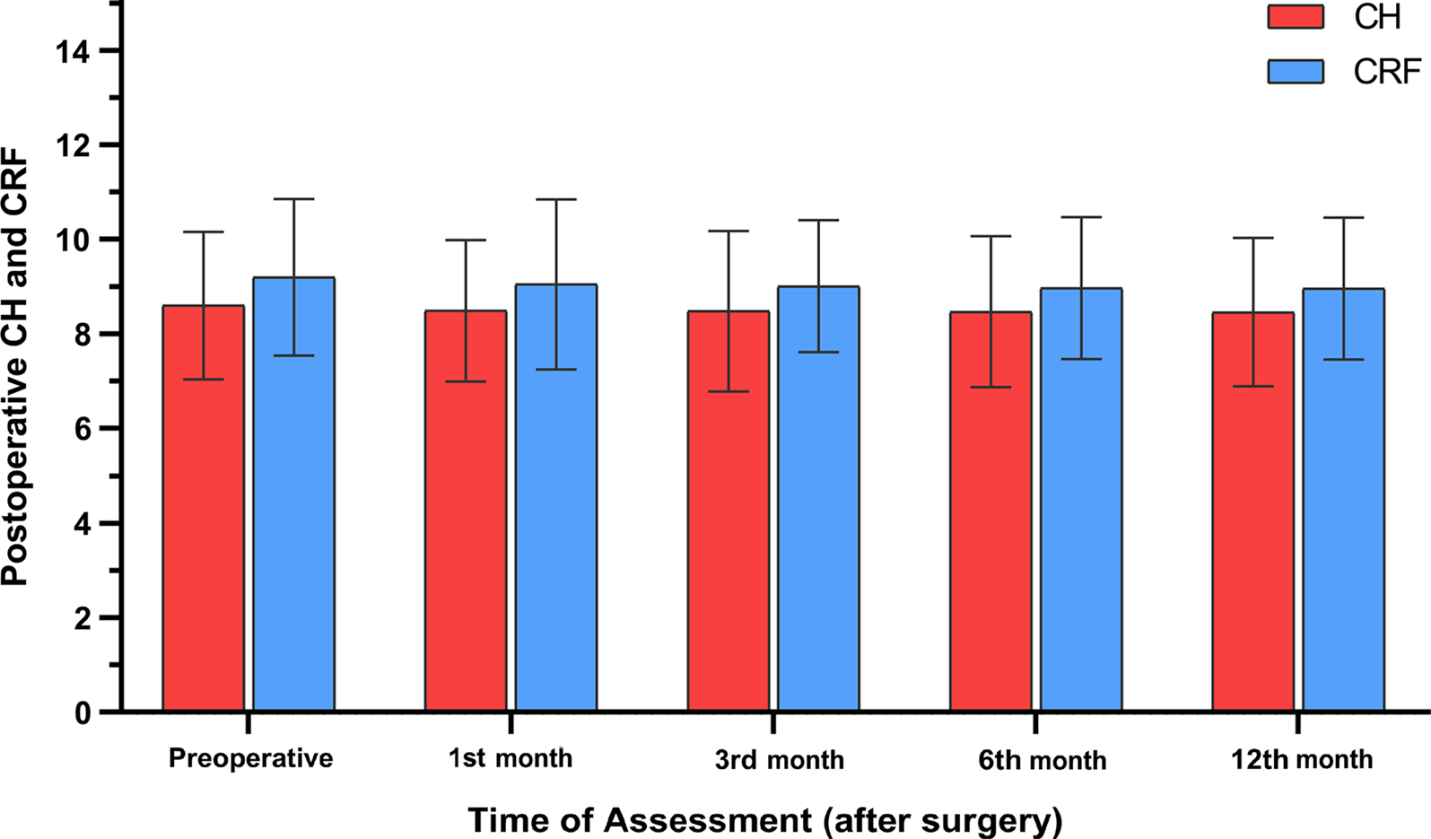
Post-TPRK changes in corneal hysteresis (CH) and corneal resistance factor (CRF)

Pre-TPRK, mean CRF was 9.20 ± 1.66 (range 8.30 to 11.50). Post-TPRK mean CRF at the 1st, 3rd, 6th, and 12th month was 9.05 ± 1.80 (range 7.38 to 10.90), 9.01 ± 1.40 (range 7.38 to 10.90), 8.97 ± 1.50 (range 7.28 to 10.90), and 8.96 ± 1.50 (range 7.29 to 10.87), respectively. No significant change in CRF was observed over the follow-up period (*P* = 0.823, Table 4 and Fig. 7).

### Complications

Corneal re-epithelialization occurred in all eyes within the 3.4 ± 0.5 days range (3 to 5 days) with no intraoperative complications. Grade 1 corneal haze was observed in 18 eyes (26.4%) and grade 2 in 4 eyes (6%) but disappeared during the first month postoperatively. Corneal haze grade 2 observed in eyes disappeared by the 3rd month postoperatively. Corneal ectasia was not detected in any of the eyes of the study. No other complications were recorded with complete healing of the epithelium within the first week postoperatively.

## Discussion

In this study, predictability, stability, efficacy, and safety of TPRK using SPT (SmartSurface procedure) of Schwind Amaris with MMC for correction of post SMILE myopic residual refractive errors were evaluated. All the treatments were done using Amaris excimer laser at 500 kHz with plano target, aspheric, and non-wavefront-guided profiles. TPRK mode has a higher laser cutting frequency than traditional PRK [13], many recent studies [14–16] have shown that this single-step TPRK has several advantages, including improved ablation algorithms and nomograms, shorter surgical time, a smaller epithelial defect than necessary for stromal ablation, no alcohol use to avoid potential limbal cell toxicity and corneal haze with shorter healing time and early visual return. The distinctive advantage of this technique is that it removes both the stroma and corneal epithelium in a single step with one ablation profile [13]. The epithelium is ablated using a custom epithelial profile created from population-based studies, revealing that the epithelium does not have a uniform thickness [17]. Besides, the biomechanics of the cornea are less affected than other refractive procedures [18]. Previous studies showed that TPRK is safe, effective, and predictable for correcting myopia and myopic astigmatism [13–19]. Also, it was used to avoid the risk of cap displacement that could theoretically occur with mechanical removal of the epithelium in patients with previous SMILE surgery [19]. It also produces a smoother and more uniform stromal bed contour than mechanical PRK, which reduces postoperative corneal haze [20]. SPT is a new Amaris laser platform software that reduces irregularities in the corneal stroma after stromal ablation, allowing for faster re-epithelization and visual recovery, particularly in the first few days after treatment with a lower incidence of postoperative corneal haze [17, 21]. MMC is used to reduce the possibility of inflammation, and subsequently, corneal haze, as any change in corneal epithelium could affect the results [19].

The retreatment rate of 3.5% reported in this study is in tandem with previous studies. Siedlecki et al. [22] reported 2.3% at the 3rd month postoperative, and Liu et al. [23] reported an incidence of 2.1% and 2.9% after 1 and 2 years, respectively, while Reinstein et al. [24, 25] reported an incidence of 4.4% in 2643 eyes after a 2-year study. In this study, there were grade 1 and grade 2 corneal haze in some cases but it was not clinically significant in terms of visual acuity, which is most likely because of the prophylactic use of MMC. Furthermore, no statistically significant differences were found between the CRF and CH values before and after TPRK. This may be because of the application of the excimer laser over the cap without creating a corneal flap.

In terms of efficacy, stability, predictability, and safety; refraction significantly improved at the 1st week and remained stable until the end of the 12th month of the follow-up time. Mean UDVA significantly improved from 0.62 to 0.1 logMAR and 100% of eyes were within ± 0.50 D of target refraction. At the 12th month post-TPRK, the efficacy index was 1.04 ± 0.17. A proportion of 14.7% of eyes gained at least one line of CDVA, 5.9% of eyes gained two lines, and CDVA remained unchanged in 79.4% of eyes (n = 54). The safety index was 1.02 ± 0.03 at the 12th month. These data are comparable to the results of surface ablation on virgin eyes. At the end of the follow-up period, the cycloplegic refraction was slightly toward the hyperopic side (mean + 0.22 ± 0.11 D). In this situation, the effects of MMC on overcorrection reported by Leccisotti [26] have to be considered.

In contrast to LASIK, which offers flap re-lifting as a safe choice for enhancement, selecting the ideal enhancement method after SMILE remains a highly debatable issue [22]. It may be theoretically easier to perform PRK or CIRCLE technique than secondary SMILE, as described by Donate and Thaeron [27]. One diopter of enhancement equals around 13 μm of the lenticule's central thickness [28]. Therefore, removing a lenticule with a thickness of less than 13 μm will be difficult without increasing the chance of breaking it, and there may not be enough anterior stroma to build another lenticule. If the primary SMILE cap interface is set at a depth of 110 ± 130 μm, the incision depth restriction makes it difficult to complete a secondary SMILE anterior to the primary procedure [26]. In this scenario, another choice would be to do a sub-cap-lenticule-extraction. The primary SMILE procedure interface becomes the superior plane of the new lenticule, and the femtosecond laser cuts only the inferior plane and the new lenticule's side-cut [27]. Another option is to create a secondary thin femtosecond laser-assisted LASIK flap (FS-LASIK) within the SMILE thick cap and then ablate [22]. This technique requires accurate preoperative assessment, including epithelial thickness measurements by high-resolution anterior segment optical coherence tomography or very high-frequency ultrasound to prevent buttonholing. It also requires residual stroma calculations to avoid a gas breakthrough between the FS-LASIK and SMILE interface [29]. Furthermore, this would cause a disproportionate effect on corneal biomechanical strength [30]. The CIRCLE technique alternative in the VisuMax software is offered, most commonly set to produce a lamellar ring at the same depth as the primary SMILE interface, a side-cut with a hinge, and an intersection cut [31]. Yet, from a patient’s viewpoint, it might appear to be puzzling to offer a flap-based retreatment option after having chosen SMILE above LASIK as a flap-free option considering its probable advantages [22]. In this perspective, TPRK offers an alternate treatment option that preserves the potential benefits of a flap-free approach. Both SMILE and TPRK have been shown to have less impact on corneal biomechanical properties [32] and tear film stability than LASIK [33]. Kling et al. [34] reported that CIRCLE technique enhancement after SMILE causes a considerably higher effect on corneal biomechanical integrity than TPRK enhancement in porcine eyes. Moreover, these results were supported by a fellow eye study in human corneas which showed that the effective elastic modulus was 1.47 times higher after SMILE than LASIK [35].

Some limitations should be considered while reading this study. In this study cohort, we have not analysed corneal aberrations and used a non-wavefront aspherical profile for all subjects. Further studies will show if some cases will benefit from corneal wavefront treatment after SMILE as reported by de Ortueta et al. [36].

## Conclusion

Transepithelial photorefractive keratectomy using smart pulse technology (SmartSurface procedure) with MMC enhancement after SMILE is a safe and effective technique with high predictability and stability that could correct post-SMILE myopic residual refractive errors and improve both CDVA and UCVA. This avoids flap-related complications caused by the manipulation of the primary SMILE cap.

## Data Availability

The datasets used and/or analysed during the current study are available from the corresponding author on reasonable request.

## Abbreviations

BSS: Balanced salt solution
CCT: Central corneal thickness
CDVA: Corrected distance visual acuity
CH: Corneal hysteresis
CRF: Corneal resistance factor
D: Diopter
IOP: Intraocular pressure
MMC: Mitomycin C
MRSE: Mean refractive spherical equivalent
ORA: Ocular response analyser
PRK: Photorefractive keratectomy
ReLEx: Refractive lenticule extraction
SE: Spherical equivalent
SD: Standard deviation
SMILE: Small incision lenticule extraction
SPSS: Statistical package for the social sciences
TPRK: Transepithelial photorefractive keratectomy
UDVA: Uncorrected distance visual acuity
